# Dynamic self-polarization of the nuclear spins in a GaAs quantum well

**DOI:** 10.1126/sciadv.aeh7568

**Published:** 2026-10-07

**Authors:** Mladen Kotur, Dennis Kudlacik, Nataliia E. Kopteva, Erik Kirstein, Dmitri R. Yakovlev, Kirill V. Kavokin, Manfred Bayer

**Affiliations:** ^1^ Experimentelle Physik 2, Technische Universität Dortmund, 44227 Dortmund, Germany.; ^2^ Spin Optics Laboratory, St. Petersburg State University, 198504 St. Petersburg, Russia.; ^3^ Research Center FEMS, Technische Universität Dortmund, 44227 Dortmund, Germany.

## Abstract

We demonstrate the self-polarization regime of nuclear spins, originally predicted in 1972 by Dyakonov and Perel and achieved here by lattice temperature reduction into the millikelvin range. We first identify a gallium arsenide-based quantum well structure as highly suited by demonstrating a high nuclear spin polarization, corresponding to an Overhauser field of 3.1 tesla, using optical spin pumping at 1.6-kelvin lattice temperature. Here, adiabatic demagnetization leads to a nuclear spin temperature of 6.4 microkelvins, detected by time-resolved Kerr rotation. By measuring polarized photoluminescence and entering the lattice temperature regime below 500 millikelvins, a sharp zero-field feature in the Hanle electron spin depolarization curve evidences the dynamic nuclear self-polarization under unpolarized optical excitation. The self-polarization results in ultralow nuclear spin temperatures, which we estimate to become as low as 200 nanokelvins.

## INTRODUCTION

In recent years, the study of the nuclear spin system (NSS) in solids has gained substantial renewed attention, largely driven by its potential application in spintronic technologies, also at the quantum level (*1*–*6*). In III-V semiconductors, such as GaAs, all of the constituent nuclei carry nonzero spin angular momentum. This gives rise to the hyperfine coupling between the spins of conduction band electrons and lattice nuclei, which represents one of the main obstacles for achieving long electron spin coherence times. A viable approach to mitigating hyperfine-induced decoherence is to suppress the nuclear spin fluctuations by inducing magnetic order in the NSS (*7*). Because nuclear spins do not interact directly with light, the NSS disorder is typically reduced through nuclear spin polarization initiated by the hyperfine interaction with optically polarized electrons (*8*, *9*). Furthermore, due to the weak coupling of the NSS to the lattice, spin-polarized nuclei generally exhibit long spin relaxation times, making them attractive for a long-lived quantum memory (*10*, *11*). Thus, a thorough understanding of nuclear spin polarization and relaxation is essential for both fundamental and application studies.

The state of an interacting NSS can be characterized by its nuclear spin temperature (*8*, *12*), which describes the population distribution of the nuclear spin states. Dynamic polarization of the nuclear spins via the hyperfine interaction with optically oriented electrons allows the system to be prepared in a low-temperature state (*13*). The nuclear spin temperature can be further reduced by adiabatic demagnetization. Here, the initial polarization of the nuclear spins determines how much the spin temperature can ultimately be reduced (*14*). This method provides an effective pathway for deep nuclear spin cooling, potentially down to submicrokelvin temperatures for which a transition to a phase with long-range magnetic order (*15*, *16*) or formation of a nuclear magnetic polaron (*17*, *18*) was predicted.

To compare, in metals, the nuclear spins can be precooled by conduction electrons to millikelvin temperatures. Then, adiabatic demagnetization can be used to further lower the spin temperature of the nuclei by a reduction factor defined by the ratio of initial and final magnetic fields in the adiabatic cooling process (*19*). In contrast, nuclear spin cooling by optical orientation in semiconductors does not require any precooling. Instead, the nuclear spins are cooled via dynamic polarization by optically oriented electron spins. The success in achieving deep nuclear spin cooling by optical pumping is largely dependent on the choice of a semiconductor structure that enables a high degree of optical spin orientation and offers long nuclear spin-lattice relaxation times. Lattice temperature reduction also plays an important role for suppressing electron spin relaxation via the spin-orbit interaction.

Along these lines, it was theoretically predicted that at lattice temperatures below 1 K a large nuclear spin polarization in weak magnetic fields may arise even under unpolarized optical pumping, so that no spin angular momentum is injected (*20*). The effect was called the dynamic self-polarization of the nuclear spins (DSPNS).

While circularly polarized excitation enables the spin transfer of the electrons to the nuclei, so that their polarization could be termed stimulated, in case of unpolarized excitation, the phenomenon of nuclear polarization is spontaneous. In both cases, the system is driven out of equilibrium, but, in the latter case, during energy relaxation, an imbalance of the nuclear spin population is spontaneously formed, which stabilizes itself by self-polarization as result of internal feedback. The emerging effective magnetic field of the nuclei (Overhauser field) acts back onto the electrons through which it can be detected.

Here, we demonstrate nuclear self-polarization in a GaAs-based quantum well (QW) in two steps: (i) demonstration of a very high stimulated nuclear polarization of 70% after circularly polarized excitation, evidencing the efficiency of dynamic polarization of the nuclear spins by electrons in the studied structure. This is important for the spontaneous imbalance of nuclear spin polarization to amplify and thereby stabilize. (ii) On this basis, we turn to helicity-modulated excitation, which is equivalent to unpolarized excitation, to prevent stimulated and allow spontaneous nuclear polarization only. In combination with cooling to subkelvin lattice temperatures, we find unique signatures for a dynamic nuclear self-polarization out of a spontaneously formed polarization. Note that this observation is achieved at close to zero external magnetic field so that there is no initial nuclear polarization.

This remarkable self-polarization effect of the nuclear spins has so far not been observed experimentally, to the best of our knowledge. Demonstrating it is a disruptive finding for the spin physics in solids because it shows that driving a spin system out of equilibrium (in this case, by unpolarized optical excitation) is the ultimate origin of spin cooling: Under nonequilibrium conditions, magnetic ordering can appear spontaneously.

In more detail, we study an undoped GaAs/(Al,Ga)As QW that demonstrates efficient electron-to-nuclear spin transfer and long nuclear spin relaxation times (*21*), making it ideally suited for deep nuclear spin cooling. Two spectroscopic techniques are used for monitoring the effect of dynamic polarization of the nuclear spins on the electron spins: time-resolved Kerr rotation (TRKR) in an oblique external magnetic field as well as optical orientation using polarized photoluminescence after circularly polarized laser excitation.

The photoluminescence technique allows us to study the behavior of the electron-nuclear spin system also at subkelvin lattice temperatures, down to 300 mK. At this temperature, we observe a peculiarity in the photoluminescence polarization dependence as a function of an applied transverse magnetic field around zero field, which indicates the spontaneous onset of nuclear spin polarization. These findings are in line with a theoretical model developed to interpret the observed effect as dynamic self-polarization of nuclear spins. We reach nuclear spin temperatures as low as 200 nK, about six orders of magnitude below the bath temperature.

## RESULTS

The photoluminescence spectrum of the studied 19.7-nm-thick GaAs/Al_0.35_Ga_0.65_As QW measured at $T_{s}=500$ mK is shown in Fig. 1A. It consists of the exciton (X) and trion (X^−^, i.e., negatively charged exciton) emission lines with maxima at 1.5272 and 1.5260 eV, respectively (*21*). The presence of the trion line, despite the sample being nominally undoped, indicates the presence of a small concentration of resident electrons of $n_{e}\leq 10^{10}\;{\text{cm}}^{-2}$. Figure 1A also shows the spectral dependence of the optical orientation $P_{\text{oo}}$, measured for circularly polarized excitation $\sigma^{+}/\sigma^{-}$ modulated at 50 kHz. At the exciton emission energy of 1.527 eV $P_{\text{oo}}$ reaches a value of about 17%.

**Fig. 1.**
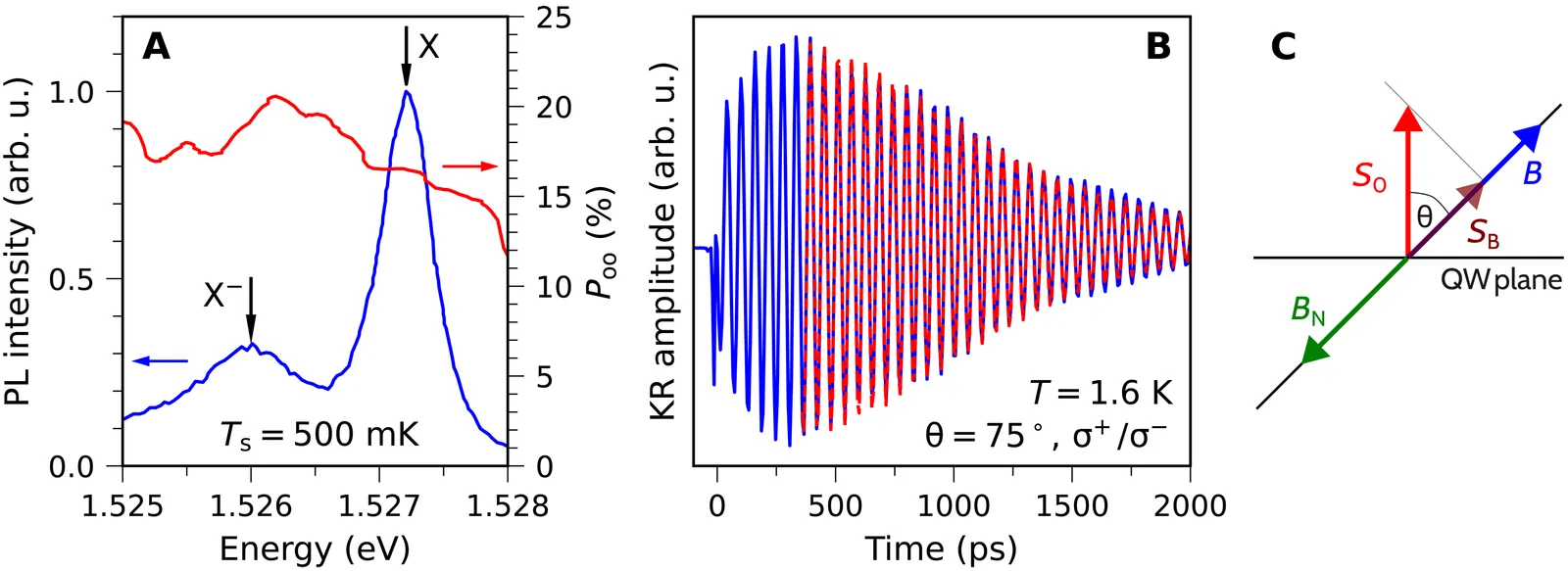
Optical orientation and coherent spin dynamics of electrons in the 19.7-nm-wide GaAs/Al_0.35_Ga_0.65_As QW. (**A**) Photoluminescence spectrum (blue line) measured at $T_{s}=500$ mK, which is temperature measured in close proximity to the sample (see Materials and Methods). The laser photon energy $E_{\text{exc}}=1.535$ eV using an excitation power P=0.1 mW. X and X^−^ denote the exciton and trion emission lines. The red line shows the spectral dependence of the optical orientation degree. (**B**) TRKR signals measured at T=1.6 K in a magnetic field of B=3 T tilted by θ=75° relative to the QW normal. The pump beam is modulated between $\sigma^{+}$ and $\sigma^{-}$ polarization at 50-kHz frequency. The pump photon energy $E_{\text{pump}}=1.527$ eV with a power of $P_{\text{pump}}=10$ mW. The red line is a fit using eq. S2. (**C**) Scheme of DNP under circularly polarized excitation in a tilted magnetic field. $S_{0}$ is the mean electron spin created by the circularly polarized pump, and $S_{B}=S_{0}\text{cos}\theta$ is the spin projection along the external magnetic field.

### Nuclear cooling detected via TRKR at 1.6 K

The hyperfine interaction can lead to spin transfer from optically oriented electrons to nuclear spins, a process referred to as dynamic nuclear polarization (DNP) (*8*, *9*, *22*). The polarized nuclear spins generate an effective magnetic field acting back on the electrons, known as the Overhauser field **B**_N_. The effect of the Overhauser field on the electron spins can be monitored by measuring the degree of circular polarization of the photoluminescence (*22*) or by the Kerr rotation of a linearly polarized probe beam (*23*). In a geometry with the magnetic field tilted relative to the light propagation direction, the nuclear spins are polarized proportionally to the projection of the mean spin of electrons on the external magnetic field; see Fig. 1C. The Overhauser field of the spin-polarized nuclei is collinear with the external magnetic field **B**, and the electron spins are exposed to the combined action of the external and Overhauser fields. The sign of the Overhauser field is determined by the helicity of the exciting light ($\sigma^{+}$ or $\sigma^{-}$). Thus, for a fixed helicity, the Overhauser field can either enhance the external field, when the two fields are parallel, or reduce it, when they are antiparallel.

We use TRKR (*24*) to study the DNP and measure the Overhauser field through monitoring its effect on the coherent dynamics of electron spins. The measurements are performed at T=1.6 K, with the laser set to $E_{\text{pump}}=1.527$ eV photon energy. The pump beam is circularly polarized having a power of $P_{\text{pump}}=10$ mW (unless stated otherwise), and the probe beam is linearly polarized using a power of $P_{\text{probe}}=0.5$ mW. When the sample is excited with helicity-modulated light ($\sigma^{+}/\sigma^{-}$), the nuclei remain unpolarized. In this case, the electron spins experience only the external magnetic field. In contrast, for constant $\sigma^{+}$ pump polarization, the DNP develops so that the electrons are subject to the combined action of the external and Overhauser magnetic fields.

Figure 1B shows the Kerr rotation amplitude measured for a modulated $\sigma^{+}/\sigma^{-}$ pump in an external magnetic field of B=3 T, tilted by θ=75° from the QW normal, taken as the *z* axis. In this case, the measured Larmor precession frequency is $\omega_{L}=0.107$ rad/ps, which corresponds to the electron *g*-factor $g_{e}=-0.40$, evaluated using eqs. S1 and S2. This value is in good agreement with the one measured on a 20-nm-wide QW and is close to that of bulk GaAs, $g_{e}=-0.44$ (*25*).

For constant $\sigma^{+}$ pump polarization, where the DNP is due, the Larmor precession frequency measured at B=3 T for θ=75° increases to $\omega_{L}=0.114$ rad/ps, corresponding to a total field of $B+B_{N}=3.3$ T, equivalent to a nuclear Overhauser field of $B_{N}=0.3$ T; see Fig. 2A.

**Fig. 2.**
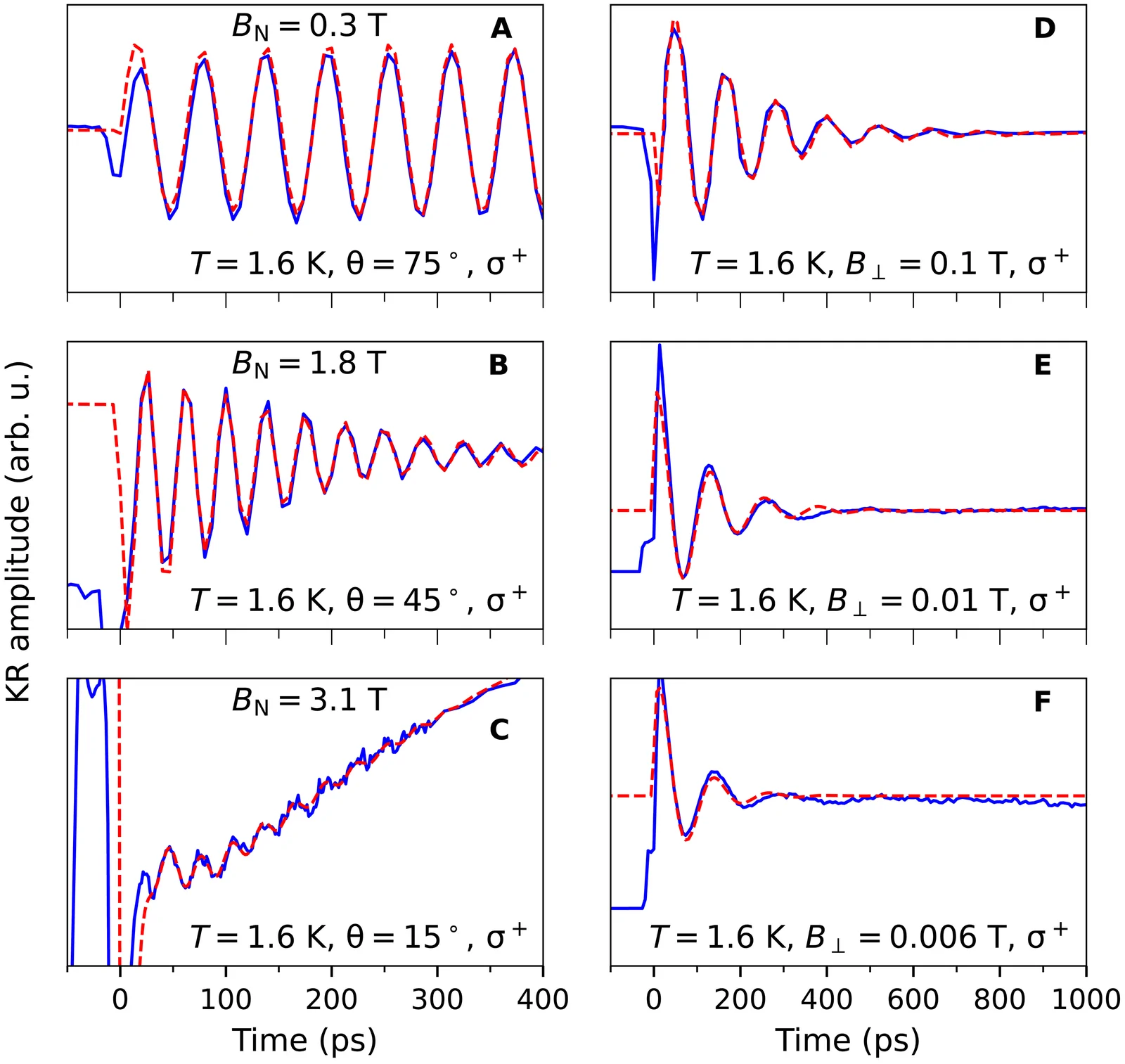
Comparison of TRKR signals measured at T=1.6 K in a tilted magnetic field of B=3 T. (**A**) θ=75°, (**B**) θ=45°, and (**C**) θ=15°. The pump beam has constant $\sigma^{+}$ circular polarization. $E_{\text{pump}}=1.527$ eV and $P_{\text{pump}}=10$ mW. The red lines are fits using eq. S2 yielding the parameters given in Table 1. (**D** to **F**) TRKR dynamics for three Voigt magnetic fields ($P_{\text{pump}}=P_{\text{probe}}=0.5$ mW). Before each measurement, the NSS was pumped for 30 min at $B_{∥}=0.6$ T ($P_{\text{pump}}=10$ mW, probe off). After pumping, the field was rotated to the Voigt geometry and reduced to the strengths given in the panels. The experimental data are shown by the solid blue lines. The dashed red lines are fits using eq. S2 with the parameters given in Table 2.

To increase the DNP and thus the Overhauser field, smaller angles of θ=45° and 15° are realized. The resulting TRKR dynamics for constant $\sigma^{+}$ pump polarization are shown in Fig. 2 (B and C). The external magnetic field is maintained at B=3 T. To minimize the effect of the linearly polarized probe beam, which could perturb the NSS via inelastic light scattering (*26*), the probe was blocked for 30 min before measuring the TRKR signal at θ=15°. The obtained Larmor precession frequencies $\omega_{L}$ and the corresponding Overhauser fields are given in Table 1.

**Table 1.** Larmor precession frequencies and corresponding Overhauser fields for various tilt angles of the external magnetic field B=3 T relative to the optical axis. $P_{N}$ is the nuclear polarization degree evaluated from the measured $B_{N}$ for $g_{e}=-0.40$ and the maximum Overhauser field of 4.3 T corresponding to $P_{N}=100\%$ (see Eq. 1).

Angle θ (°)	ω_L_ (rad/ps)	$B_{N}$ (T)	*P*_N_(%)
75	0.114	0.3	7
45	0.168	1.8	42
15	0.215	3.1	72

One can see that, for θ=15°, the Overhauser field reaches a remarkable strength of $B_{N}=3.1$ T. Note that similar or even larger values of the Overhauser field, up to 4 T, were reported in GaAs quantum dots, where the electron confinement leads to a much stronger interaction with the nuclei than in a wide QW or in bulk (*27*, *28*).

A nuclear polarization of 100% corresponds to the state in which all Ga and As nuclear spins are aligned, resulting in an electron spin splitting of $\Delta_{\text{eN}}\approx 100$ μeV (*6*). With the electron *g*-factor of $g_{e}=-0.40$, this corresponds to the maximum Overhauser field of

$$B_{N}^{\text{max}}=\frac{\Delta_{\text{eN}}}{\mu_{B}|g_{e}|}=4.3\;T$$ (1)

Therefore, the measured $B_{N}=3.1$ T corresponds to 72% of complete nuclear polarization in the studied 19.7-nm-wide GaAs QW. Considering the weak confinement in such a wide QW, this result represents an unexpectedly efficient nuclear spin polarization compared with quantum dots. This demonstration of an efficient DNP is essential for the nuclear dynamic self-polarization as it facilitates amplification of any nuclear polarization imbalance. Note that nuclear spin ordering effects in semiconductors are predicted to occur once the nuclear polarization reaches 70%, which is surpassed in our study. At this polarization level, the NSS system may undergo a phase transition into a ferromagnetic or antiferromagnetic state (*15*) upon adiabatic demagnetization to zero external field.

To demonstrate that the nuclear spin polarization follows the predictions of the spin temperature theory, we use a spin cooling procedure based on the principle of adiabatic demagnetization (*29*). The nuclear cooling begins with pumping the nuclear spins in a longitudinal magnetic field of $B_{∥}=0.6$ T for 30 min. During this time, the pump laser power is set to $P_{\text{pump}}=10$ mW, and the probe beam is blocked. After 30 min, the pump beam is also blocked, and the magnetic field is gradually rotated from the Faraday to the Voigt configuration, keeping its magnitude constant at 0.6 T. Then, the Voigt field is slowly reduced at a rate of ∼300 mT/min from its initial value $B_{⊥}\left(0\right)$ [in our case, $B_{⊥}\left(0\right)=0.6$ T] to the target value $B_{⊥}$.

The nuclear spin temperature $\Theta_{N}$ follows from a universal expression dictated by the entropy conservation during the adiabatic demagnetization

$$\Theta_{N}\left(B_{⊥}\right)=\Theta_{N}\left(0\right)\sqrt{\frac{B_{⊥}^{2}+B_{L}^{2}}{B_{⊥}{\left(0\right)}^{2}+B_{L}^{2}}}$$ (2)

Here, $\Theta_{N}\left(0\right)$ is the initial nuclear spin temperature achieved in the external magnetic field $B_{⊥}\left(0\right)$, before adiabatic demagnetization. The local magnetic field $B_{L}$, which is about 0.1 mT in bulk GaAs (*30*, *31*), characterizes the interactions within the NSS. In nanostructures, $B_{L}$ can be significantly enhanced by the quadrupole interaction due to the strain inherent to these systems (*32*). To maintain the adiabatic regime of demagnetization, the condition ${\mathrm{dB}}_{⊥}/\mathrm{dt}<B_{L}/T_{2}$ should be satisfied, which is fulfilled in our experiments. Here, $T_{2}$ is the transverse spin relaxation time, which is determined by the spin-spin interactions between the nuclei. In GaAs, it is on the order of 100 μs (*8*). Accordingly, the nuclear spin polarization $P_{N}$ can be expressed as

$$P_{N}=\frac{B_{⊥}}{3k_{B}\Theta_{N}\left(B_{⊥}\right)}\hbar \langle \gamma_{N}\left(I+1\right)\rangle$$ (3)

Here, *I* is the nuclear spin, $k_{B}$ is the Boltzmann constant, and $\gamma_{N}$ is the nuclear gyromagnetic ratio. The angular brackets denote the averaging over all nuclear species (^69^Ga, ^71^Ga, and ^75^As), weighted with their natural abundances (*29*).

When the external field is decreased during the adiabatic demagnetization to the desired target value, the pump beam with the power reduced to $P_{\text{pump}}=0.5$ mW, and the probe beam ($P_{\text{probe}}=0.5$ mW) are unblocked so that the TRKR signal can be recorded, revealing Larmor precession oscillations of the electron spins. The data obtained for $B_{⊥}=0.1$, 0.01, and 0.006 T are shown in Fig. 2 (D to F). From the extracted Larmor precession frequencies $\omega_{L}$ of 0.053, 0.05, and 0.05 rad/ps, $B_{N}$ is found to be about 1.4 T, independent of the $B_{⊥}$ strength; see Table 2. Such behavior is expected from Eqs. 3 and 2 for external magnetic fields that are much stronger than the local magnetic field, $B_{⊥}\gg B_{L}$. The nuclear spin temperatures evaluated from Eq. 3 for the three external magnetic fields $B_{⊥}=0.1$, 0.01, and 0.006 T are $\Theta_{N}=106.5$, 10.7, and 6.4 μK, respectively. It follows from Eq. 2 that, for $B_{⊥}=0$ T and $B_{L}=0.8$ mT (*21*), the nuclear spin temperature would be $\Theta_{N}=0.8$ μK. The larger value of $B_{L}$ in the QW, compared with that in bulk GaAs, where it is governed by the dipole-dipole interactions between the nuclei, arises from the additional strain inherent to micro- and nanostructures.

**Table 2.** Evaluated nuclear spin temperatures. Given parameters are received as fit for results in Fig. 2 (D to F) using eq. S2.

$B_{⊥}$ (T)	ω_L_ (rad/ps)	$B_{N}$ (T)	Θ_N_ (μK)	$T_{2}^{*}$ (ps)
0.1	0.053	1.4	106.5	190
0.01	0.05	1.4	10.7	110
0.006	0.05	1.4	6.4	70

For comparison, on the same sample, we previously measured a nuclear spin temperature of 0.54 μK using the adiabatic demagnetization method in the rotating frame (*7*). Although lower nuclear spin temperatures were reported for CdTe QWs, $\Theta_{N}=0.3$ μK (*33*), $\Theta_{N}=0.54$ μK remains, to the best of our knowledge, the lowest measured nuclear spin temperature for GaAs.

Unfortunately, due to challenges with compensating residual fields in our superconducting magnet, it was not possible to decrease the external field to strengths below 6 mT. However, the fact that the Overhauser field and the related nuclear spin polarization remain constant upon lowering the external magnetic field down to the value just above the local field of spin-spin interactions demonstrates the validity of the spin temperature concept for the conditions of our experiments. This is one of the essential prerequisites for observation of the dynamic self-polarization of nuclear spins at low external magnetic fields.

### Dynamic self-polarization of nuclear spins at 300 mK

To examine the properties of the electron-nuclear spin system at the minimal achievable lattice temperatures, where the regime of DSPNS potentially can be reached (*20*), we use the optical orientation technique. The photoluminescence was excited with circularly polarized light and its circular polarization was measured. The Hanle effect is studied, monitoring the depolarization of the luminescence by a transverse external magnetic field or by the transverse component of a tilted field. The sample was cooled in a cryogen-free dilution refrigerator with the base temperature of 10 mK without sample illumination. To minimize the laser heating of the sample, we use low excitation powers of 0.01 mW, which allows us to reach $T_{s}=300$ mK, and of 0.1 mW for $T_{s}=500$ mK.

The Hanle curves measured under helicity-modulated circular excitation ($\sigma^{+}/\sigma^{-}$) are shown in Fig. 3 for perpendicular (θ=90°) and tilted (θ=60°) magnetic fields with respect to the light wave vector **k**, at sample temperatures of 500 and 300 mK. For comparison, measurements performed at 1.6 K, where the sample is in direct contact with liquid helium, are also shown in Fig. 3 (A and D) for θ=90° and 70°.

**Fig. 3.**
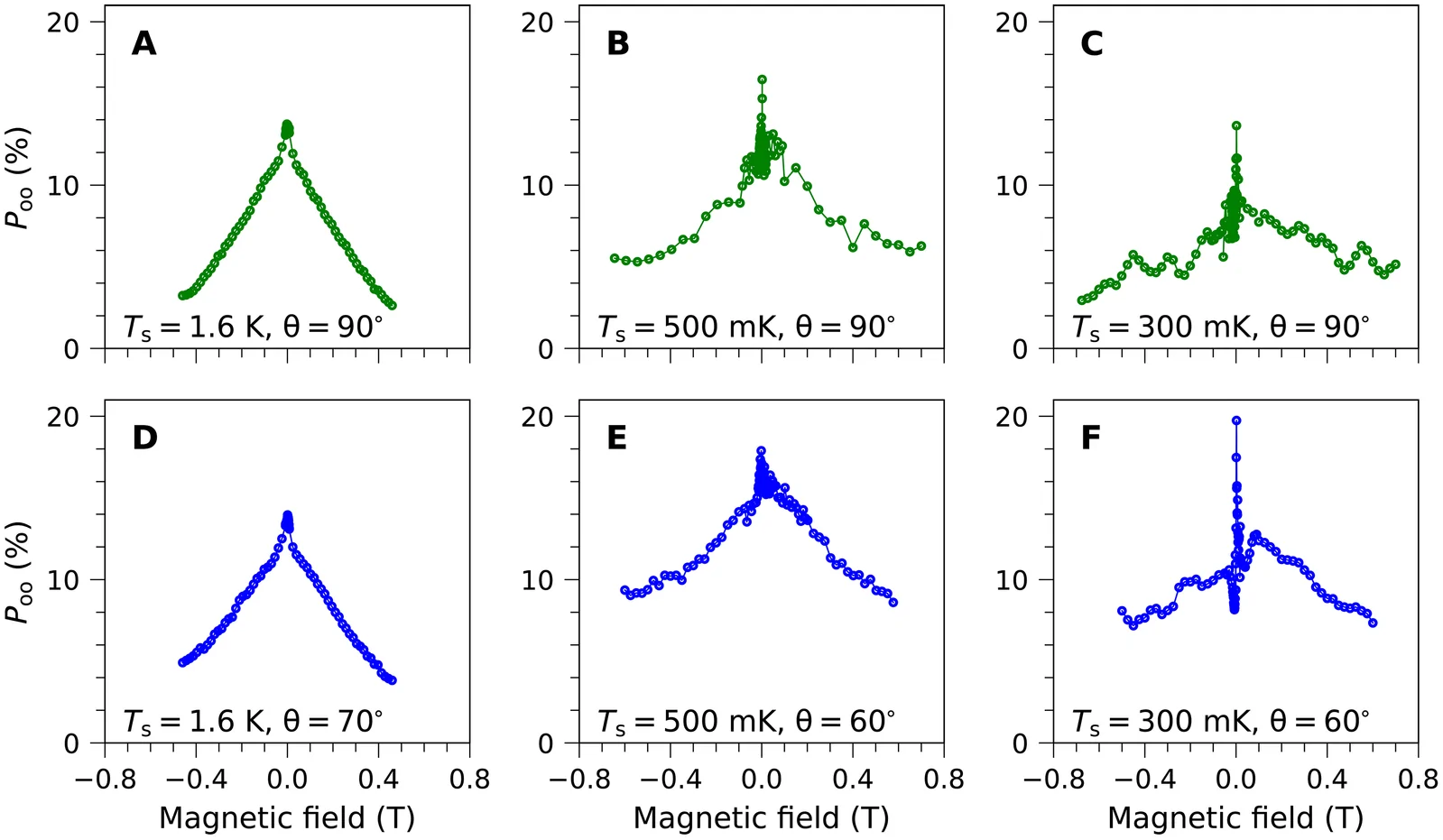
Hanle effect measured at various temperatures. Hanle curves measured in Voigt magnetic field (θ=90°) under polarization-modulated, $\sigma^{+}/\sigma^{-}$ at 50 kHz, excitation with $E_{\text{exc}}=1.535$ eV for (**A**) $T_{s}=1.6$ K and P=0.5 mW, (**B**) $T_{s}=500$ mK and P=0.1 mW, and (**C**) $T_{s}=300$ mK and P=0.01 mW. Hanle curves measured in a tilted magnetic field geometry under modulated $\sigma^{+}/\sigma^{-}$ excitation for (**D**) $T_{s}=1.6$ K, P=0.5 mW, and θ=70°; (**E**) $T_{s}=500$ mK, P=0.1 mW, and θ=60°; and (**F**) $T_{s}=300$ mK, P=0.01 mW, and θ=60°.

The main feature of the Hanle curves, which appears when cooling the structure below 1 K, is the narrow peak at zero magnetic field. When comparing the data at 300 and 500 mK, one can see that the optical orientation degree ($P_{\text{oo}}$) at B=0 does not change significantly, but the wings at finite magnetic fields decrease strongly with decreasing temperature, so that the narrow peak becomes much more pronounced. The exact orientation of the magnetic field does not seem to have a large impact.

The emergence of a zero-field peak is usually considered as fingerprint of the dynamic polarization of nuclear spins by spin-polarized electrons. It reflects the buildup of an Overhauser field, which becomes possible when the external field overcomes the local fields of the nuclear spin-spin interactions (*8*, *9*). However, in our case, the electron spin polarization is alternated at high frequency, with a period much shorter than both the spin-spin and the spin-lattice relaxation times of the nuclei (20 μs versus ∼0.1 ms and 100 s, respectively). Under these conditions, spin polarization cannot accumulate in the NSS (*8*), so that, from perspective of the nuclei, the exciting light is in effect unpolarized as are the electrons regarding their interaction with the nuclear spins. See the detailed considerations of this issue in sections S4 and S5. The circular polarization of the exciting light is only important for measurement of the Hanle effect of the electron spin polarization.

Dynamic polarization of nuclei by unpolarized photoexcited electrons via the Overhauser effect (*8*) is possible at low temperatures if an external magnetic field is applied to the structure. The Overhauser effect occurs when the hyperfine interaction provides the main channel of spin relaxation of electrons. If the applied field is strong enough to induce a considerable equilibrium magnetization of electrons at the given lattice temperature, then the electrons can relax to this equilibrium spin-polarized state by flip-flop transitions involving nuclear spins. In this process, nuclei will acquire spin polarization opposite to that of the electrons because the hyperfine interaction conserves the total angular momentum of the interacting particles. If the electron spin ensemble is maintained unpolarized by an external agent like microwave radiation, optical excitation, or electric injection, then the flip-flop transitions would provide an uninterrupted spin flow into the NSS. As the spin-lattice relaxation time of the nuclei at low lattice temperatures is typically very long, nuclear spin polarization will accumulate, reaching values of the order of the equilibrium spin polarization of electrons, which can be considerable at subkelvin lattice temperatures and external magnetic fields of the order of 1 T. However, in our experiments, the external field in the vicinity of the zero-field peak of the Hanle curve is, by far, not enough to provide high nuclear polarization by the classical Overhauser effect.

In 1972, Dyakonov and Perel suggested that the Overhauser effect may occur in the effective magnetic field of polarized nuclear spins (the Overhauser field) and theoretically predicted DSPNS at temperatures below ∼1 K (*20*). The essence of the effect is as follows. Suppose that the nuclei spontaneously attain some spin polarization, parallel or antiparallel to the weak external magnetic field, while the electron spins are initially unpolarized. Because the hyperfine interaction in GaAs is antiferromagnetic (positive hyperfine constant), the equilibrium state of electrons at low lattice temperature acquires nonzero mean spin opposite to that of the nuclei (we neglect here the Zeeman interaction of the electrons with the weak external field). Because the electrons initially are not spin polarized, the magnitude of their mean spin will increase toward the equilibrium value. If the main channel of electron spin relaxation is provided by the hyperfine interaction, then they will take the required angular momentum from the NSS. As a result, the nuclei will acquire additional spin polarization opposite to the equilibrium polarization of the electrons, i.e., parallel to the initial polarization of the nuclei, which therefore will increase. If the electrons are maintained unpolarized by optical excitation, then this process will repeat over and over again, and the NSS will reach a considerable spin polarization (see the diagram in Fig. 4A). Note that the only influence of the external field in this effect is to stabilize the spin polarization of the nuclei; the nuclear spins can develop polarization either parallel or antiparallel to the external field, depending on the direction of the initial nuclear spin fluctuation.

**Fig. 4.**
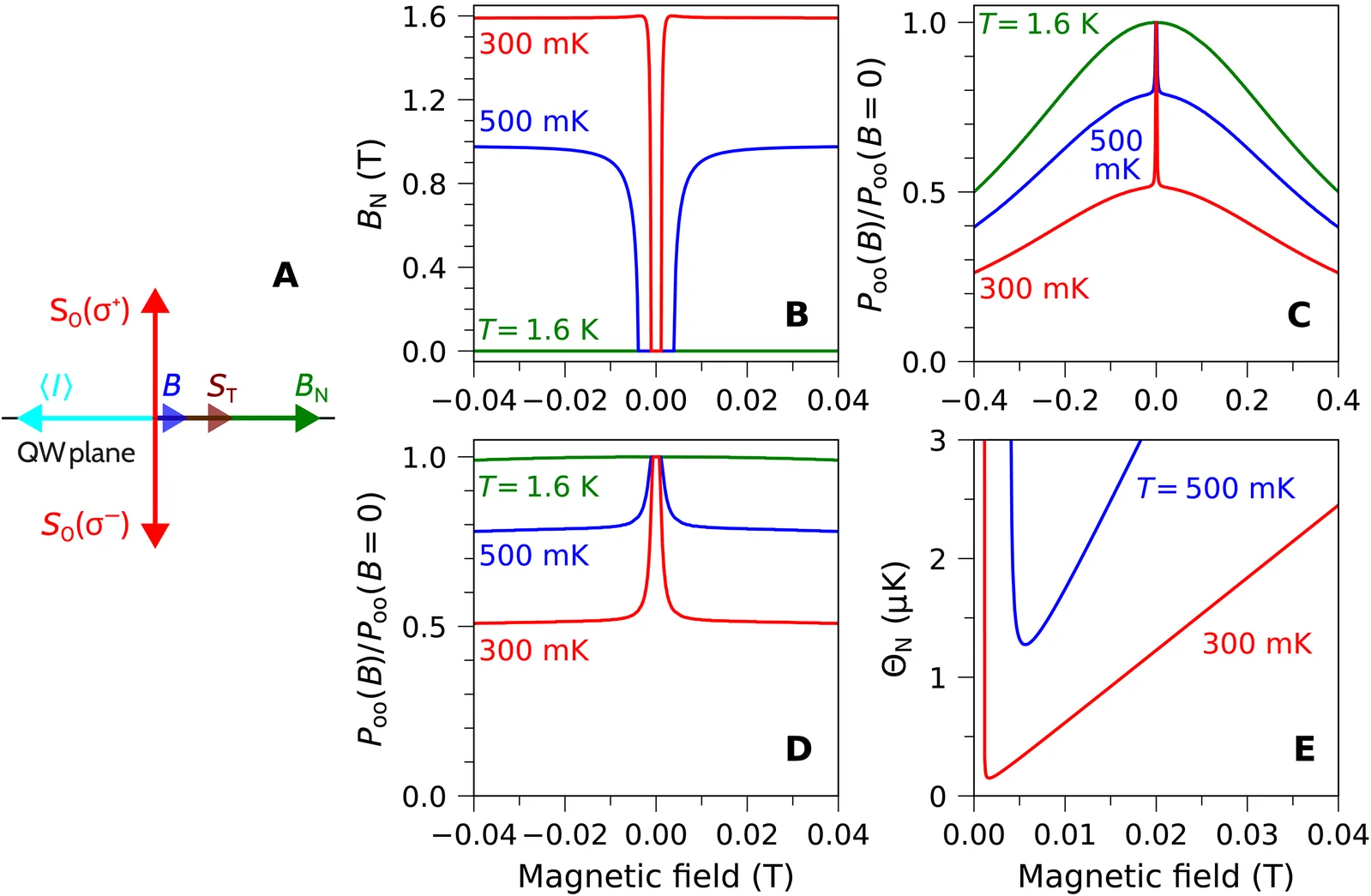
Modeling of the DSPNS. (**A**) Diagram explaining the self-polarization of nuclear spins under helicity-modulated optical excitation, maintaining zero electron spin projection onto the external field. Here, $S_{0}\left(\sigma^{+}\right)$ and $S_{0}\left(\sigma^{-}\right)$ are the mean electron spins created during the $\sigma^{+}$ and $\sigma^{-}$ half-cycle of the helicity modulation. $S_{T}$ is the thermal equilibrium mean spin of the electrons in the nuclear field $B_{N}$. 〈I〉 is the mean spin of the nuclei arising in the process of electron spin relaxation toward $S_{T}$. (**B** to **E**) Calculated characteristics of the electron-nuclear spin system of the 19.7-nm-wide GaAs/Al_0.35_Ga_0.65_As QW in the regime of DSPNS, as functions of the external magnetic field *B* in Voigt geometry (θ=90°). (B) Overhauser field $B_{N}$ as function of the external magnetic field for T=1.6 K, 500 mK, and 300 mK, calculated using eq. S13 with the following parameters: ${\tilde{B}}_{L}=1.4$ mT, f=0.55, and $B_{N}^{\text{max}}=4.3$ T. (C) Normalized PL polarization as a function of the external magnetic field at various temperatures (*f* covering the range from 0 to 1 with the maximum at 0.26). (D) Zoom into (C) in the magnetic-field range of ±0.04 T. (E) Dependence of the nuclear spin temperature $\Theta_{N}$ on the external magnetic field for 500- and 300-mK bath temperature, calculated for f=0.55.

In section S3, we reconsider the Dyakonov and Perel model for the conditions of our experiment. The theory predicts the onset of DSPNS upon fulfillment of two conditions: (i) The lattice temperature is below the critical temperature $T_{c}$ defined by the condition $k_{B}T_{c}=5f\mu_{B}g_{e}B_{N}^{\text{max}}/6$, where f<1 is the leakage factor determined by the ratio of the nuclear relaxation rates through the hyperfine interaction with carriers and through other [presumably, quadrupole-induced; (*32*)] relaxation mechanisms; and (ii) the external magnetic field is larger than the critical field $B_{c}=B_{L}\sqrt{3T/\left(T_{c}-T\right)}$.

One can see from the calculations in Fig. 4B that, once the self-polarization conditions are reached, the Overhauser field rapidly grows to values of about 1 T for T=500 mK and 1.6 T for T=300 mK. This results in the sharp drop in the optical orientation degree dependence on the transverse external magnetic field, as shown in Fig. 4C. This drop occurs when the external magnetic field *B* reaches $B_{c}$ in absolute value, leading to the zero-field peak in the Hanle curve, which is shown in more detail in Fig. 4D. Qualitatively, this behavior resembles the experimental observations, and, therefore, the DSPNS effect is the natural explanation of the observed features in the Hanle curves presented in Fig. 3 (C and F). Note that the DSPNS in low external fields (just above $B_{c}$) results in lowering of the nuclear spin temperature $\Theta_{N}$ by 6 orders of magnitude, down to 200 nK at T=300 mK (Fig. 4E).

However, the DSPNS theory faces some challenges in the quantitative description of our experimental data. First, the value of f≈0.26, estimated from the oblique field Hanle effect (fig. S3) yields a value of $T_{c}$ slightly smaller than 300 mK so that DSPNS is not expected to develop at this value of *f*. Second, with the half width at half maximum of the Hanle curve of 0.4 mT and the Overhauser field of the order of 1 T, which is predicted to develop once DNSPS occurs, one would expect a polarization drop to almost zero, which is not observed in Fig. 3 (C and F).

A possible explanation is an inhomogeneity of the leakage factor *f*. Assuming that *f* is statistically distributed in the range from 0 to 1 with the maximum probability near 0.26 and averaging the PL polarization in the transverse geometry over *f* yield the Hanle curves shown in Fig. 4C, which match the experimental curves in Fig. 3 (C and F).

## DISCUSSION

It is worth to summarize the required conditions to reach in the multidimensional parameter space the sweet spot for observing the DSPNS effect: (i) the presence of charge carriers (either resident or photoexcited) coupled by the hyperfine interaction to the spins of the lattice nuclei; (ii) optical excitation with unpolarized, linearly polarized, or high-frequency modulation-polarized light maintains zero time-averaged polarization of the electron spins; also, any other external perturbation causing average carrier spin depolarization could be applied; (iii) lattice temperature below some critical value (typically, in the subkelvin range), which depends on the leakage factor determined by the ratio of rates of nuclear relaxation by the hyperfine interaction with carriers and by other mechanisms; and (iv) external magnetic field above a certain critical value (typically, of the order of 1 mT) determined by the local fields of the nuclear spin-spin interactions and the temperature; the field direction can be arbitrarily oriented, the nuclear spins will polarize along this field. As all these conditions are satisfied in our experiments, we are convinced that the observation of the sharp zero-field feature in the Hanle curve upon lowering the temperature below 1 K has to be interpreted as manifestation of the dynamic self-polarization of nuclear spins, predicted theoretically by Dyakonov and Perel in 1972 (*20*).

We would like to note that, although DSPNS was claimed to be experimentally observed in (*34*, *35*), those experiments used NMR detection and, correspondingly, were performed in strong magnetic fields. Therefore, while the experimental conditions might allow for DSPNS, the detected nuclear spin polarization was not spontaneous but was actually induced by the external field via the Overhauser effect. The Overhauser effect could be enhanced by the nuclear field adding to the external field, which provides a high degree of nuclear spin polarization, but the key feature of DSPNS, the spontaneous onset of nuclear spin polarization in weak magnetic fields close to the local field of the nuclear spin-spin interaction, has not been demonstrated.

It is worth to mention other mechanisms of nuclear self-polarization that were theoretically proposed for narrow QWs or quantum dots. They are based on nonlinearities arising because of shifts of the electron spin levels in the Overhauser field, which affect the efficiency of quantum transitions involved in the optical excitation and relaxation of excitons (*36*, *37*) or electron tunneling in coupled quantum dots (*38*). Although those proposals do not require low lattice temperatures, they have not yet been experimentally realized, to the best of our knowledge.

As we mentioned above for the conditions of the DSPNS effect, any external perturbation providing depolarization of the average carrier spin is suitable. One may think on using electrical injection to provide unpolarized electrons and realize the dynamic self-polarization of the nuclei. Still one would need a technique to detect the nuclear spin polarization. To that end, optical orientation is the most straightforward method. A combination of electric injection with optical detection may be an interesting strategy to reduce optical heating and reach lower temperatures.

The presented results demonstrate that a wide undoped GaAs QW can sustain strong nuclear polarization and deep nuclear cooling, approaching the regime where collective nuclear-spin ordering is expected, paving the way for future studies of ordered nuclear-spin phases in semiconductors.

## MATERIALS AND METHODS

### Sample

The sample was grown by molecular-beam epitaxy on a Te-doped GaAs substrate. It consists of 13 nominally undoped GaAs/Al_0.35_Ga_0.65_As QWs separated by 30.9-nm-thick barriers. The widths of the QWs range from 2.8 to 39.3 nm. This sample was previously investigated in (*7*, *21*, *39*, *40*). Here, we report results for the QW with width of 19.7 nm.

### Time-resolved Kerr rotation

A mode-locked Ti:sapphire laser has pulse duration of 1.5 ps (spectral width of about 1 meV) and repetition rate of 76 MHz. The laser beam was split into the circularly polarized pump and the time delayed linearly polarized probe. The pump polarization was either kept constant, $\sigma^{+}$, using a λ/4 wave plate or modulated $\sigma^{+}/\sigma^{-}$ by a photoelastic modulator (PEM) at a frequency of 50 kHz. The reflected probe beam was detected using a balanced photodiode behind a polarization optics for Kerr rotation analysis. The TRKR setup is schematically shown in fig. S1. For the TRKR experiments, the sample was mounted in a bath cryostat being at temperature of T=1.6 K in direct contact with superfluid helium. The cryostat has a superconducting vector magnet allowing for fields up to 3 T in any direction. The magnetic field **B** was applied either in the Faraday geometry (θ=0°, $B_{∥}∥k$), the Voigt geometry (θ=90°, $B_{⊥}⊥k$), or at an intermediate tilt angle θ between these two geometries [θ=∠(B,k)]. Here, k is the wave vector of the pump laser, aligned with the sample growth axis (*z* axis).

### Optical orientation

To photogenerate spin-polarized electrons, the sample was excited with circularly polarized light from a Ti:sapphire laser tuned to the photon energy of $E_{\text{exc}}=1.535$ eV. The helicity of the circular polarization was either modulated between $\sigma^{+}$ and $\sigma^{-}$ at a frequency of 50 kHz using PEM or kept fixed at $\sigma^{+}$ by means of a quarter-wave (λ/4) plate.

The PL was detected using an avalanche photodiode connected to a photon counter, following spectral dispersion by a monochromator; see fig. S2. The intensities of the $\sigma^{+}$ ($I^{+}$) and $\sigma^{-}$ ($I^{-}$) circularly polarized emission were recorded, allowing us to measure the optical orientation degree: $P_{\text{oo}}=\left(I^{+}-I^{-}\right)/\left(I^{+}+I^{-}\right)$ (*8*, *9*). The magnetic field dependence of $P_{\text{oo}}$ (Hanle curves) was measured for magnetic fields applied either in the Voigt geometry (θ=90°, $B_{⊥}⊥k$) or in tilted fields.

For experiments at temperatures below 1.6 K, the sample was mounted on a copper puck thermally anchored to the mixing chamber plate of a cryogen-free dilution refrigerator (Oxford Instruments Proteox), with the base temperature of T=10±2 mK reached without sample illumination. The cryostat is equipped with optical windows allowing for direct optical excitation and detection. The temperature was monitored using two calibrated sensors, one positioned on the mixing plate ($T_{m}$) and another one placed on the sample holder in close proximity to the sample ($T_{s}$). The cryostat is equipped with a superconducting vector magnet, generating fields up to $B_{z}=5$ T in the Faraday geometry and up to $B_{x}=B_{y}=1$ T in the Voigt geometry.

## Supplementary Materials

### The PDF file includes:

Sections S1 to S6 Figs. S1 to S4 Table S1 Legend for data S1 References
