# Supplementary material for: Dynamic self-polarization of the nuclear spins in a GaAs quantum well
Source: Sci Adv. 2026 Oct 7;12(41):eaeh7568. doi: 10.1126/sciadv.aeh7568 (PMC13644773; doi:10.1126/sciadv.aeh7568)
Supplement: Supplementary file 1 [file sciadv.aeh7568_sm.pdf]

Science Advances
AAAS

Supplementary Materials for

Dynamic self-polarization of the nuclear spins in a GaAs quantum well

Mladen Kotur *et al.*

Corresponding author: Mladen Kotur, mladen.kotur@tu-dortmund.de;
Dmitri R. Yakovlev, dmitri.yakovlev@tu-dortmund.de

Sci. Adv. **12**, eaeh7568 (2026)
DOI: 10.1126/sciadv.aeh7568

The PDF file includes:

Sections S1 to S6
Figs. S1 to S4
Table S1
Legend for data S1
References

Other Supplementary Material for this manuscript includes the following:

Data S1

S1. METHODS

Figure 1A presents the PL spectrum of the 19.7-nm-wide QW measured at $T_s = 500$ mK, as indicated by the temperature sensor at the sample. Although the base temperature in our cryostat is about 10 mK, optical excitation causes heating of the sample, which is monitored with a sensor placed in close proximity. As a result, a laser power of 0.1 mW is used to provide a compromise between limiting the sample heating, keeping the temperature in the millikelvin range, and maintaining a sufficient signal-to-noise ratio. The maximum intensity of the PL at 1.527 eV, attributed to exciton (X) emission, was chosen as the PL detection energy for all subsequent measurements.

Time-resolved Kerr rotation

The experimental setup used for measuring time-resolved Kerr rotation is shown in Figure S1.

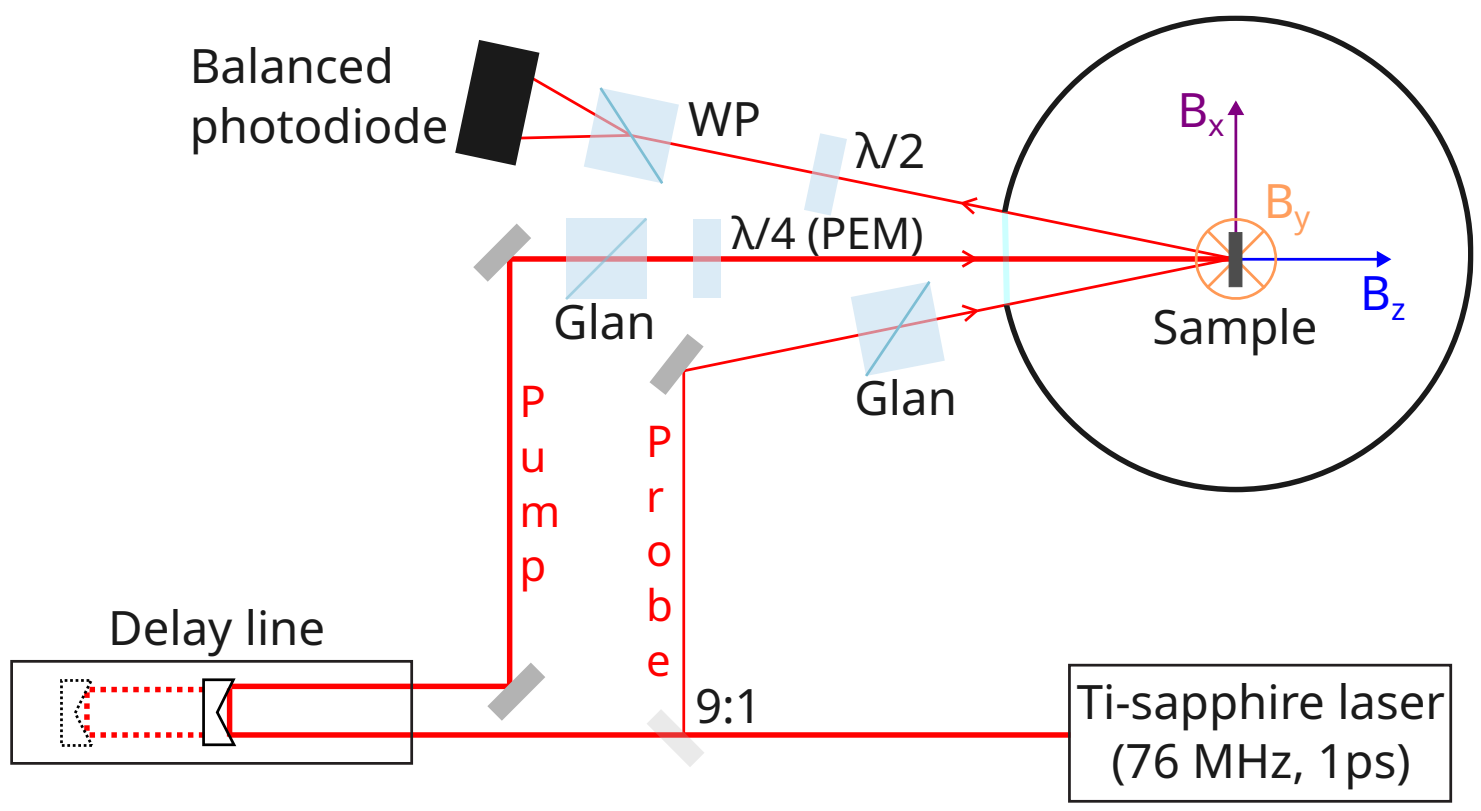

FIG. S1. **Time-resolved Kerr rotation setup.** The magnetic field components B_x, B_y and B_z indicate field orientations corresponding to the Voigt (B_x) and Faraday (B_z) geometries. For a circularly polarized pump with constant σ^+ helicity, a $\lambda/4$ wave plate was used, which was replaced by a photoelastic modulator (PEM) for helicity modulated σ^+/σ^- excitation.

For the TRKR experiments the sample was mounted in the variable-temperature insert of a liquid helium bath cryostat, allowing operation from 1.6 K up to room temperature. At $T = 1.6$ K the sample was in direct contact with superfluid helium. The cryostat was equipped with a superconducting vector magnet allowing for fields up to 3 T in any direction. In our experiments, the magnetic field $\mathbf{B}$ was applied either in the Faraday geometry ($\theta = 0°$, $\mathbf{B}_\parallel \parallel \mathbf{k}$), the Voigt geometry ($\theta = 90°$, $\mathbf{B}_\perp \perp \mathbf{k}$), or at an intermediate tilt angle θ between these two geometries ($\theta = \angle(\mathbf{B}, \mathbf{k})$). Here, $\mathbf{k}$ is the wave vector of the pump laser, aligned with the sample growth axis (z-axis).

TRKR [24] was used to study the nuclear spin polarization and evaluate the Overhauser field via its influence on the electron spins. Excitation with a circularly polarized pump pulse generates electron spins oriented along the light propagation axis. Applying the external magnetic field in the tilted geometry causes the initial spin polarization to precess about $\mathbf{B}$ at the Larmor precession frequency

$$\omega_L = \frac{|g_e|\mu_B B}{\hbar}\,. \tag{S1}$$

Here g_e is the electron g-factor, μ_B is the Bohr magneton, and $\hbar$ is the reduced Planck constant. A time-delayed, linearly polarized probe pulse monitors the evolution of the spin projection along the optical axis, S_z, via the Kerr rotation angle θ_{KR}, since $\theta_{\mathrm{KR}} \propto S_z$. For the analysis of the TRKR signal, the data were fitted using the following equation, describing the Larmor precession about the external magnetic field [9]

$$\theta_{\mathrm{KR}}(t) = A_{\mathrm{KR}} \exp\left(-t/T_2^*\right) \cos(\omega_L t). \tag{S2}$$

Here, A_{KR} is the amplitude of the TRKR signal, and T_2^* is the electron spin dephasing time.

The TRKR experimental setup is schematically shown in Fig. S1. A mode-locked Ti:Sapphire laser with a repetition rate of 76 MHz provided the pulsed excitation, with a pulse duration of 1.5 ps and a spectral half-width at half-maximum of about 1 meV. The laser beam was split into the circularly-polarized pump and the time delayed linearly-polarized probe. The pump polarization was either kept constant, σ^+, using a $\lambda/4$ wave plate or modulated σ^+/σ^- by a photoelastic modulator (PEM) at a frequency of 50 kHz. The reflected probe beam was detected using a balanced photodiode behind a polarization optics, composed of an achromatic half-wave plate and a Wollaston prism (WP) for Kerr rotation analysis.

Dynamical nuclear polarization induced by optically-oriented carriers and detected via polarized photoluminescence

The experimental setup used for measuring the dynamic nuclear polarization under continuous-wave excitation is shown in Figure S2.

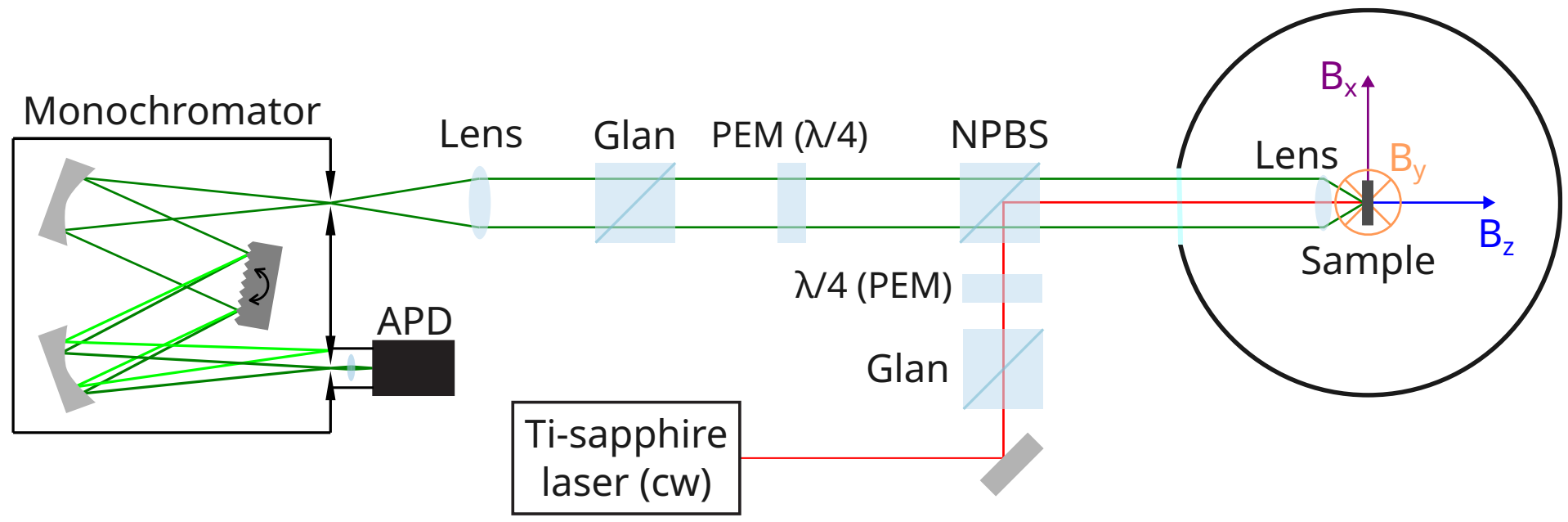

FIG. S2. **Setup for DNP and optical nuclear cooling using continuous-wave (cw) laser excitation.** NPBS is a non-polarizing beam splitter. APD is an avalanche photodiode. The magnetic field components B_x, B_y, and B_z indicate field orientations that correspond to the Voigt (B_x) and Faraday (B_z) geometries.

For the DNP and nuclear cooling experiments at temperatures below 1.6 K, the sample was mounted on a copper puck thermally anchored to the mixing chamber plate of a cryogen-free dilution refrigerator (Oxford Instruments Proteox), with the base temperature of $T = 10 \pm 2$ mK reached without sample illumination. The cryostat is equipped with optical windows allowing for direct optical excitation and detection. The temperature was monitored using two calibrated sensors, one positioned on the mixing plate (T_m) and another one placed on the sample holder in close proximity to the sample (T_s). The cryostat is equipped with a superconducting vector magnet, generating fields up to $B_z = 5$ T in the Faraday geometry and up to $B_x = B_y = 1$ T in the Voigt geometry. A lens was placed inside the puck right next the sample and aligned with the optical axis of the window, see Fig. S2. It was used to focus the excitation laser beam onto the sample and to collect the photoluminescence (PL).

To photogenerate spin-polarized electrons, the sample was excited with circularly polarized light from a Ti:Sapphire laser tuned to $E_{exc} = 1.535$ eV photon energy. The helicity of the circular polarization was either modulated between σ^+ and σ^- at a frequency of 50 kHz using a photoelastic modulator (PEM), or kept fixed at σ^+ by means of a quarter-wave ($\lambda/4$) plate. In the first case, the rapid alternation of the electron spin orientation suppresses dynamic nuclear polarization. Fixed σ^+ excitation maintains a constant electron spin orientation, allowing DNP to develop. The PL was detected using an avalanche photodiode connected to a photon counter, following spectral dispersion by a monochromator. The polarization components were analyzed using the circular polarization analyzer in the detection channel, consisting of a PEM (for σ^+ excitation) or a $\lambda/4$ wave-plate (for modulated σ^+/σ^- excitation) and a Glan-Taylor prism. The PEM was synchronized with the photon counter to enable polarization-resolved detection. The intensities of the σ^+ (I^+) and σ^- (I^-) circularly polarized emission were recorded, allowing us to measure the optical orientation degree: $P_{\text{oo}} = (I^+ - I^-)/(I^+ + I^-)$ [8, 9]. The magnetic field dependence of P_{oo} (Hanle curves) was measured for magnetic fields applied either in the Voigt geometry ($\theta = 90^\circ$, $\mathbf{B}_\perp \perp \mathbf{k}$) or in tilted fields.

S2. NUCLEAR SPIN COOLING DETECTED BY OPTICAL ORIENTATION OF ELECTRONS AT 500 mK

In order to examine DNP and optical cooling of the nuclear spin system at temperatures below 1.6 K, we use the optical orientation technique monitoring the polarization of the photoluminescence. Namely, we study the Hanle effect, which describes the depolarization of the luminescence by a transverse external magnetic field [41], or, in our case, by the transverse component of the field applied at a tilt angle. If the sample is excited with helicity modulated light (σ^+/σ^- at 50 kHz), due to the rapid changes of the electron spin orientation, the nuclei remain unpolarized [8]. In this case the electron spin is influenced only by the external magnetic field and the Hanle curve is symmetric with respect to magnetic field inversion.

The Hanle curve measured under helicity modulated excitation at a temperature of $T_s = 500$ mK ($T_m = 45$ mK) is shown in Fig. S3, as indicated by the blue circles. The external magnetic field $\mathbf{B}$ is applied at a tilt angle of $\theta = 60^\circ$. Due to the absence of an Overhauser field, the Hanle curve exhibits symmetry relative to zero field. The Hanle curve changes considerably for σ^+ excitation (red circles in Fig. S3), with a shoulder appearing at positive magnetic fields

having its maximum at 0.16 T. At this magnetic field, the Overhauser field exactly compensates the external field, therefore, one can evaluate $B_N = -0.16$ T. The steady-state Overhauser field B_N^{st}, i.e., the effective magnetic field generated by the nuclei once their polarization has reached equilibrium under optical pumping, is given by [21, 42]

$$B_N^{st} = B_N^{max} \frac{I+1}{S(S+1)} f S_0 \cos\theta. \tag{S3}$$

Here, $B_N^{max} = 4.3$ T is the maximum Overhauser field defined by Eq. (1), and S_0 is the mean electron spin. Since we detect the PL emission due to recombination of electrons having spin projection on the QW axis equal to $\pm 1/2$ with heavy holes, S_0 can be evaluated as $S_0 = P_{oo}/2$. The parameter f in Eq. (S3) is the leakage factor, defined as

$$f = \frac{T_L}{T_{1e} + T_L}, \tag{S4}$$

where T_{1e} and T_L denote the nuclear spin relaxation times through electrons and through all other channels, respectively. The leakage factor effectively quantifies the efficiency of the dynamic nuclear polarization driven by optically-oriented electrons. Since in our case $|B_N^{st}| = 0.16$ T (corresponding to $P_N = 0.037$) and $\theta = 60°$, it follows from Eq. (S3) that the leakage factor is $f \approx 0.26$. It is worth noting that the nuclear spin polarization of $P_N = 0.037$ is achieved at a very low excitation power of $P = 0.1$ mW, which we kept low to keep the temperature in the millikelvin range. Increasing the excitation power would increase P_N and, consequently, the Overhauser field B_N, but it would also cause lattice heating, which has the opposite effect on P_N. For comparison, in our previous work [39] we were able to reach Overhauser field values close to 0.6 T (corresponding to $P_N = 0.14$) at $T = 1.8$ K, but with a much higher excitation power of $P = 10$ mW.

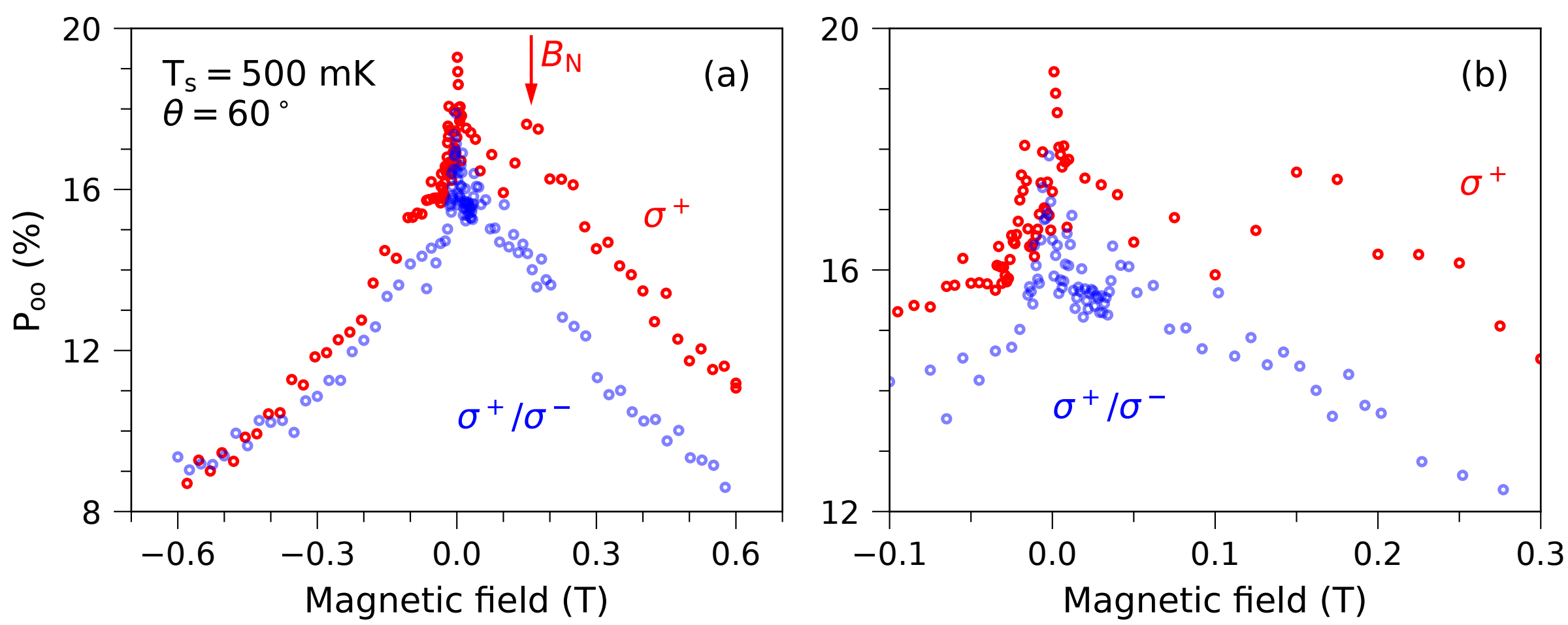

FIG. S3. **Hanle effect measured at $T_s = 500$ mK.** (a) Hanle curves in a magnetic field tilted by $\theta = 60°$, measured with modulated σ^+/σ^- (blue circles) and constant σ^+ (red circles) excitation helicity at $T_s = 500$ mK. $E_{exc} = 1.535$ eV with $P = 0.1$ mW. The arrow indicates the strength of the Overhauser field of the spin polarized nuclei $B_N = -0.16$ T. (b) Enlarged view of the same data over a smaller magnetic-field range.

To gain deeper insight into the collective nuclear spin behavior, we proceeded with investigating the nuclear spin dynamics under illumination. To this end, we followed a protocol analogous to those described in Refs. [43, 44]. In the first stage, the circularly polarized (σ^+) excitation light is blocked for 500 s using a mechanical shutter, and no external magnetic field is applied. This ensures that, at the beginning of the second stage, there is no nuclear spin polarization, as the spins relax during the dark period with a characteristic spin-lattice relaxation time T_1, where $T_1^{-1} = T_{1e}^{-1} + T_L^{-1}$. For the 19.7-nm-wide QW, the relaxation time T_1 during optical pumping depends on temperature and is about 400 s at $T = 10$ K [21], decreasing to just above 200 s at $T = 1.8$ K [39], see also Table S1. In contrast, at $T = 10$ K, in darkness and at zero magnetic field, T_1 is only a few seconds [21]. Therefore, the chosen 500 s dark period is sufficiently long to ensure that no nuclear polarization has remained at the start of the second stage.

Just before the end of the first stage, a tilted magnetic field of 0.16 T is applied at an angle of $\theta = 60°$. The laser is then unblocked, and the optical orientation degree is recorded as a function of time. The measured P_{oo} dynamics are shown in Fig. S4. It can be clearly seen that the degree of optical orientation increases with time before reaching saturation. The reason for this dynamics is that during excitation the nuclear spins become dynamically polarized, resulting in the emergence of an Overhauser field. This field compensates the external magnetic field, reducing the

TABLE S1. Comparison of the nuclear spin-lattice relaxation time measured at $T_s = 500$ mK under illumination with previously reported values, recorded at Kelvin-range temperatures for the 19.7-nm-wide $GaAs/Al_{0.35}Ga_{0.65}As$ QW.

Temperature (K)	Relaxation time T_1 (s)
0.5	150
1.8	210 [39]
6	300 [39]
10	450 [39]
10	~ 400 [21]
20	~ 300 [21]

effective field on the electrons and thereby increasing the degree of optical orientation. The time dependence of the Overhauser field is given by [21]

$$B_N(t) = B_N^{st}[1 - \exp(-t/T_1)]. \tag{S5}$$

Thus, by fitting the $P_{oo}(t)$ dynamics with an exponential function $\sim \exp(-t/T_1)$, we obtain the T_1 time under illumination. The experimental value extracted from Fig. S4 is $T_1 = 150$ s. This time has to be compared with previously measured values on the same sample at temperatures ranging from 1.8 to 20 K, summarized in Table S1. For temperatures above 6 K, the relaxation time T_1 remains temperature-independent, despite some scatter of the experimental data. As reported in Ref. [21], the dominant relaxation mechanism of nuclear spins is hyperfine scattering on free photoexcited two-dimensional electrons. This process is described theoretically in Ref. [45], where the spin relaxation rate for nondegenerate free electrons in a QW of width d was derived as

$$\frac{1}{T_{1,e}} \propto \frac{A^2\Omega^2 n_e m^*}{\hbar^3 d^2}. \tag{S6}$$

Here, A denotes the hyperfine constant, Ω represents the unit cell volume, n_e is the two-dimensional electron density, and m^* is the electron effective mass.

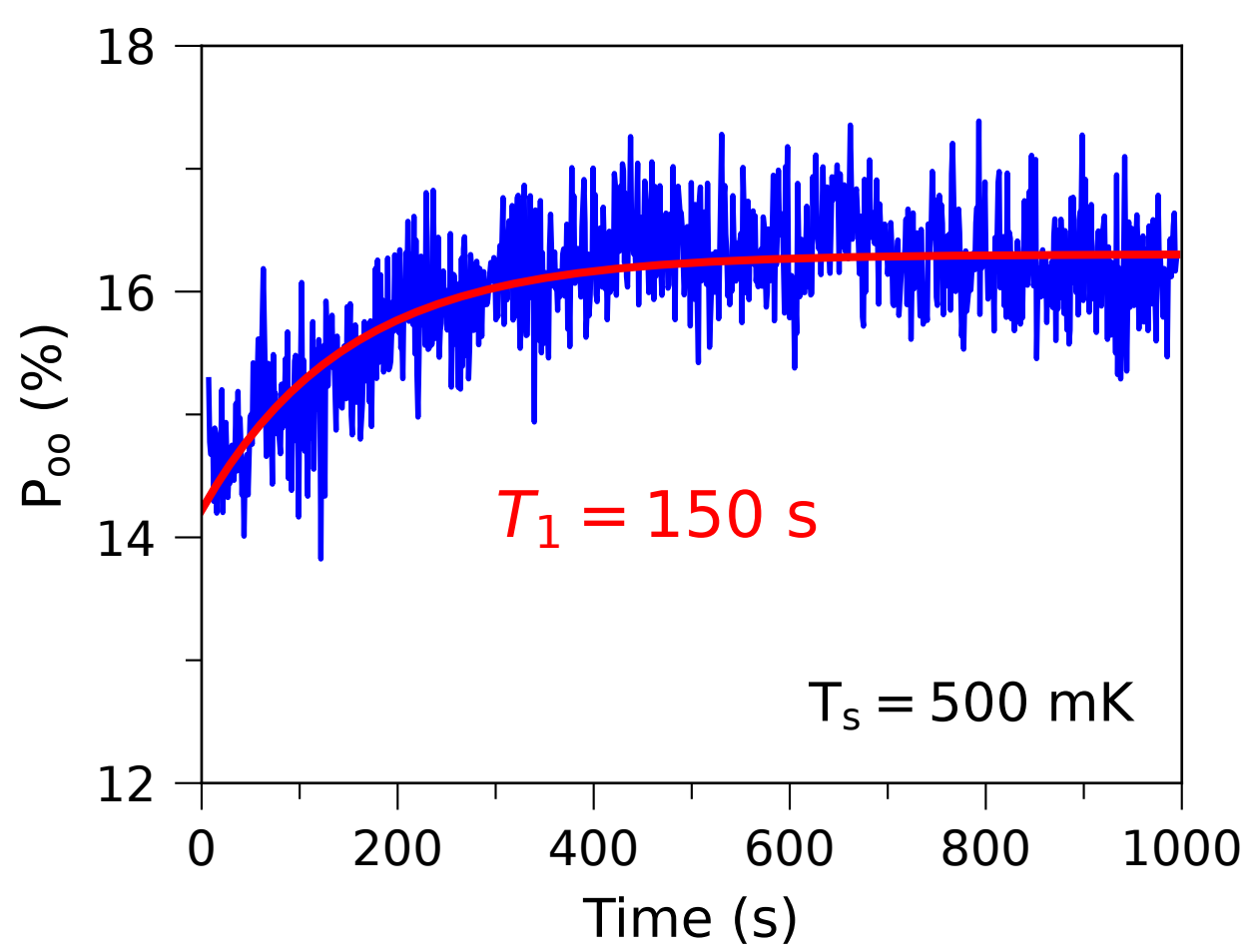

FIG. S4. **Dynamics of the nuclear polarization.** Build-up of the nuclear spin polarization measured through the change in the optical orientation degree after starting the illumination of the sample at time zero, following a 500 s dark period, in a magnetic field of $B = 0.16$ T tilted by $\theta = 60°$. The sample temperature is $T_s = 500$ mK. The excitation photon energy $E_{exc} = 1.535$ eV with a power $P = 0.1$ mW. The red line shows an exponential fit, from which the nuclear spin polarization build-up time $T_1 = 150$ s is evaluated.

Equation (S6) indicates that the spin relaxation time $T_{1,e}$ should remain invariant with respect to temperature. However, at temperatures below 6 K, it is observed to decrease as the temperature is lowered. A plausible explanation for the T_1 temperature dependence below 6 K is the onset of electron localization [39]. In this regime, localized electrons, owing to their longer correlation time, transfer their spin to the nuclei more efficiently than free electrons do, thereby accelerating the nuclear spin relaxation [8].

S3. THEORY OF THE DYNAMIC SELF-POLARIZATION OF NUCLEAR SPINS

Here, we reconsider the Dyakonov and Perel model [20] for the conditions of our experiment, and show that the DSPNS effect is the only plausible explanation of the observed features in the Hanle curves in Figs. 3C,F.

It was demonstrated previously that the spin polarization of the lattice nuclei in GaAs follows the spin-temperature theory both at low [29] and high magnetic fields [46]. Within the spin-temperature approach, the Overhauser field $\mathbf{B}_N$ acting on the electrons is parallel to the external magnetic field $\mathbf{B}$ and its value is determined by the nuclear spin temperature Θ_N

$$B_N \approx B_N^{max} \mathrm{B}_{3/2}\left(\frac{\hbar\bar{\gamma}_N B}{k_B \Theta_N}\right). \tag{S7}$$

Here, $\mathrm{B}_{3/2}$ is the Brillouin function for spin $I = 3/2$, $\bar{\gamma}_N$ is the nuclear gyromagnetic ratio averaged over the isotopes, and B_N^{max} is the Overhauser field for full polarization of the nuclear spins. The spin temperature of the nuclei coupled by the hyperfine interaction to photoexcited electrons is given by [13]

$$\frac{1}{k_B\Theta_N} = \frac{4f}{\hbar\bar{\gamma}_N}\frac{B}{B^2+\tilde{B}_L^2}\frac{\bar{S}_B - S_T}{1-4\bar{S}_B S_T} \approx -\frac{4f}{\hbar\bar{\gamma}_N}\frac{B}{B^2+\tilde{B}_L^2}S_T. \tag{S8}$$

Here, $\tilde{B}_L \approx \sqrt{3}B_L$ [13] is the characteristic field of the nuclear spin-spin interactions, $\bar{S}_B$ is the projection of the mean electron spin on the external field $\mathbf{B}$ (on average, close to zero under modulated pumping, see for details Secs. S4 and S5 below) and f is the leakage factor. S_T is the thermodynamic equilibrium value of the electron spin projection on $\mathbf{B}$, determined by the total field acting onto the electron spins and by the lattice temperature T

$$S_T = -\frac{1}{2}\mathrm{B}_{1/2}\left(\frac{\mu_B g_e (B_N + B)}{k_B T}\right) = -\frac{1}{2}\tanh\left(\frac{\mu_B g_e (B_N+B)}{2k_B T}\right). \tag{S9}$$

Combining Eqs. (S7), (S8), and (S9), one arrives at an equation for the Overhauser field B_N

$$B_N = B_N^{max}\mathrm{B}_{3/2}\left[\frac{2B^2 f}{B^2+\tilde{B}_L^2}\mathrm{B}_{1/2}\left(\frac{\mu_B g_e (B_N+B)}{k_B T}\right)\right]. \tag{S10}$$

In order to analyze the behavior of the Overhauser field near zero external field, one can neglect B as compared to B_N in Eq. (S10). Assuming that $\mu_B g_e B_N \ll k_B T$, which is reasonable for our experimental conditions up to $B_N \sim 1$ T, we expand the Brillouin function up to the 3-rd power of the argument $\mathrm{B}_I(x) \approx \frac{1}{3}I(I+1)x - \frac{1}{90}I(I+1)[2I(I+1)-1]x^3$. These simplifications result in a 3-rd order algebraic equation for $z = \mu_B g_e B_N / k_B T$

$$\left(\frac{T}{T_0}\right) z = \frac{5}{6}\frac{B^2 f}{B^2+\tilde{B}_L^2} \left\{ z - \left[\frac{1}{24} + \frac{17}{30}\left(\frac{B^2 f}{B^2+\tilde{B}_L^2}\right)^2\right] z^3 \right\}, \tag{S11}$$

where $T_0 = \mu_B g_e B_N^{max}/k_B$. The value of $\Delta_{eN} = \mu_B g_e B_N^{max}$, which is the Overhauser splitting of the electron spin levels for full polarization of the nuclear spins, was recently measured experimentally in GaAs [6] and approximately equals to 100 μeV, yielding $B_N^{max} = 4.3$ T and $T_0 \approx 1.16$ K.

Equation (S11) has the trivial solution $z_0 = 0$, corresponding to the absence of nuclear polarization. However, if the temperature is lower than $T_c = \frac{5}{6} f T_0$, which is equal to 0.95 K in case of low leakage (where $T_{1e} \ll T_L$ and $f = 1$), two other solutions appear in external magnetic fields exceeding the critical field $B_c = \tilde{B}_L\sqrt{\frac{T}{T_c - T}}$:

$$z_{\pm} = \pm\sqrt{\frac{1 - \frac{6}{5}\frac{B^2+\tilde{B}_L^2}{B^2 f}\left(\frac{T}{T_0}\right)}{\frac{1}{24}+\frac{17}{30}\left(\frac{B^2 f}{B^2+\tilde{B}_L^2}\right)^2}}. \tag{S12}$$

Besides, it can be shown that these states are stable, while z_0 loses stability. These solutions correspond to a non-zero Overhauser field, equal in absolute value to

$$B_{Nsp}(B,T) = B_N^{max}\left(\frac{T}{T_0}\right)|z_{\pm}| = B_N^{max}\left(\frac{T}{T_0}\right)\sqrt{\frac{1 - \frac{6}{5}\frac{B^2+\tilde{B}_L^2}{B^2 f}\left(\frac{T}{T_0}\right)}{\frac{1}{24}+\frac{17}{30}\left(\frac{B^2 f}{B^2+\tilde{B}_L^2}\right)^2}}. \tag{S13}$$

The emergence of these solutions manifests the onset of the DSPNS effect.

S4. COMMENT ON THE DIFFERENCE BETWEEN UNPOLARIZED EXCITATION AND EXCITATION WITH LIGHT OF HELICITY ALTERNATED AT HIGH FREQUENCY

This issue is discussed in detail in Ref. 47. The helicity-alternated light creates both an oscillating spin flow into the nuclear spin system and an oscillating hyperfine field (Knight field) at the nuclear spins. Their combination results in two opposite energy flows between the electrons and the nuclear spin system: one due to the injection of magnetic moment into the NSS subject to the Knight field, and the other due to the action of the time-dependent Knight field on the nuclear magnetization created by the spin injection previously. The net energy flow is proportional to the Fourier component of the time derivative of the nuclear spin correlation function at the frequency of helicity modulation, which equals unity at zero frequency and vanishes when the frequency becomes much larger than the inverse spin-spin relaxation time. As a result, excitation by light with the helicity alternated at a high frequency does not change the NSS energy and, therefore, does not result in nuclear spin cooling. An important exception from this rule is the effect of resonant cooling of the NSS, observed if the helicity modulation frequency coincides with the Larmor frequency of the nuclear spins in the applied transverse magnetic field [48].

Since the derivation of this important result in Ref. 47 is not quite transparent and contains misprints in key equations, we re-derive it below in Sec. S5.

Another possible effect of the helicity-modulated excitation on the NSS is heating of the previously cooled nuclear spins by the oscillating Knight field. This effect manifests itself in experiments on all-optical nuclear magnetic resonance [48]. However, if the modulation frequency is far from the NMR resonance frequency (as is the case when no external magnetic field is applied), this effect is also negligible.

To summarize, excitation with helicity-alternated light may exert an effect on the NSS only if the modulation frequency is close to the NMR frequency in the applied external field. In our case, it could have appeared at fields around 5 mT, however we do not observe any features on the Hanle curves around this field. No effect of the helicity-alternated excitation is expected at fields below and around 1 mT, where the onset of nuclear polarization is observed. At low external field, the helicity-alternated excitation we use is equivalent to unpolarized excitation.

S5. DERIVATION OF THE COOLING RATE BY HELICITY-ALTERNATED LIGHT

Spin flip-flop transitions result in angular momentum transfer to the NSS with the rate $j_S = \hbar\bar{\gamma}_N T_{eN}^{-1} S_z(t)$, where T_{eN}^{-1} is the rate of electron spin relaxation by the hyperfine interaction with nuclear spins. In presence of the oscillating Knight field $B_e(t) = b_e S_z(t)$ the NSS energy changes with the rate

$$j_E(t) = j_S(t) B_e(t) + M_N(t)\frac{dB_e(t)}{dt}, \tag{S14}$$

where

$$M_N(t) = \int_0^\infty j_S(t-\tau)G(\tau)d\tau + \int_0^\infty B_e(t-\tau)\alpha(\tau)d\tau \tag{S15}$$

is the magnetic moment of the NSS arising as result of dynamic polarization by electrons (first term) and NSS magnetization under the Knight field (second term). Here $G(\tau)$ is the correlation function of the non-equilibrium nuclear spin, while $\alpha(\tau)$ is the magnetic susceptibility of the NSS. The first term in Eq. (S14) is the energy injection from the electrons, accompanying the dynamic spin polarization in presence of the Knight field. If S_z oscillates with the angular frequency ω, $S_z = S_0\cos(\omega t)$, Eq. (S14) rewrites as

$$\begin{aligned} j_E(t) &= \hbar\bar{\gamma}_N T_{eN}^{-1} S_0 b_e \left[\cos^2(\omega t) - \omega\sin(\omega t)\int_0^\infty \cos[\omega(t-\tau)]G(\tau)d\tau\right] - b_e^2 S_0^2\omega\sin(\omega t)\int_0^\infty \cos[\omega(t-\tau)]\alpha(\tau)d\tau \\ &= \hbar\bar{\gamma}_N T_{eN}^{-1} S_0 b_e \left[\cos^2(\omega t) - \omega\sin(\omega t)\cos(\omega t)\int_0^\infty \cos(\omega\tau)G(\tau)d\tau - \omega\sin^2(\omega t)\int_0^\infty \sin(\omega\tau)G(\tau)d\tau\right] \\ &\quad - b_e^2 S_0^2\left[\omega\sin(\omega t)\cos(\omega t)\int_0^\infty \cos(\omega\tau)\alpha(\tau)d\tau + \omega\sin^2(\omega t)\int_0^\infty \sin(\omega\tau)\alpha(\tau)d\tau\right]. \end{aligned} \tag{S16}$$

When averaging over the helicity oscillation period, the terms containing $\sin(\omega t)\cos(\omega t)$ vanish. As a result we obtain for the average energy rate

$$\begin{aligned} \bar{j}_E &= \frac{1}{2}\hbar\bar{\gamma}_N T_{eN}^{-1} S_0 b_e\left[1 - \omega\int_0^\infty \sin(\omega\tau)G(\tau)d\tau\right] - \frac{1}{2}b_e^2 S_0^2\omega\int_0^\infty \sin(\omega\tau)\alpha(\tau)d\tau \\ &= -\frac{1}{2}\hbar\bar{\gamma}_N T_{eN}^{-1} S_0 b_e\int_0^\infty G'(\tau)\cos(\omega\tau)d\tau - \frac{1}{2}b_e^2 S_0^2\omega\alpha''(\omega), \end{aligned} \tag{S17}$$

where $\alpha''(\omega)$ is the imaginary part of the NSS susceptibility at the frequency ω, and $G'(\tau) = dG(\tau)/d\tau$. In obtaining Eq. (S17), we took the integral in square brackets in Eq. (S16) by parts, taking into account that $G(0) = 1$. The last term in Eq. (S17) describes the NSS warm-up by the oscillating Knight field. The first term, responsible for the NSS cooling by dynamic polarization in the Knight field, is proportional to the Fourier component of $G'(\tau)$, that vanishes at high frequencies. For this reason, no cooling occurs at modulation frequencies much higher than the spectral width of the nuclear spin correlation function, which can be estimated as T_{SS}^{-1}, where T_{SS} is the nuclear spin-spin relaxation time. Since $G(\tau = 0) = 1$ and $G(\tau \to \infty) \to 0$, at zero frequency

$$\int_0^\infty G'(\tau)\cos(\omega\tau)d\tau = \int_0^\infty G'(\tau)d\tau = -1, \tag{S18}$$

and as a result

$$\bar{j}_E = \frac{1}{2}\hbar\bar{\gamma}_N T_{eN}^{-1} S_0 b_e\,. \tag{S19}$$

S6. DATA FOR FIGURES

In the file Data S1.zip data for figures are included in CSV format.

REFERENCES

1. I. Žutić, J. Fabian, S. Das Sarma, Spintronics: Fundamentals and applications. *Rev. Mod. Phys.* **76**, 323–410 (2004).

2. B. Urbaszek, X. Marie, T. Amand, O. Krebs, P. Voisin, P. Malentinsky, A. Högele, A. Imamoglu, Nuclear spin physics in quantum dots: An optical investigation. *Rev. Mod. Phys.* **85**, 79–133 (2013).

3. D. Gangloff, G. Éthier-Majcher, C. Lang, E. Denning, J. Bodey, D. Jackson, E. Clarke, M. Hugues, C. Le Gall, M. Atatüre, Quantum interface of an electron and a nuclear ensemble. *Science* **364**, 62–66 (2019).

4. D. M. Jackson, D. A. Gangloff, J. H. Bodey, L. Zaporski, C. Bachorz, E. Clarke, M. Hugues, C. Le Gall, M. Atatüre, Quantum sensing of a coherent single spin excitation in a nuclear ensemble. *Nat. Phys.* **17**, 585–590 (2021).

5. M. T. Uysal, M. Raha, S. Chen, C. M. Phenicie, S. Ourari, M. Wang, C. G. Van de Walle, V. V. Dobrovitski, J. D. Thompson, Coherent control of a nuclear spin via interactions with a rare-earth ion in the solid state. *PRX Quantum* **4**, 010323 (2023).

6. P. Millington-Hotze, H. E. Dyte, S. Manna, S. F. C. Silva, A. Rastelli, E. A. Chekhovich, Approaching a fully-polarized state of nuclear spins in a solid. *Nat. Commun.* **15**, 985 (2024).

7. M. Kotur, D. O. Tolmachev, V. M. Litvyak, K. V. Kavokin, D. Suter, D. R. Yakovlev, M. Bayer, Ultra-deep optical cooling of coupled nuclear spin-spin and quadrupole reservoirs in a GaAs/(Al,Ga)As quantum well. *Commun. Phys.* **4**, 193 (2021).

8. F. Meier, B. P. Zakharchenya, Eds., *Optical Orientation* (North-Holland, 1984).

9. M. I. Dyakonov, Ed., *Spin Physics in Semiconductors* (Springer International Publishing AG, 2017).

10. W. M. Witzel, S. Das Sarma, Nuclear spins as quantum memory in semiconductor nanostructures. *Phys. Rev. B* **76**, 045218 (2007).

11. J. J. L. Morton, A. M. Tyryshkin, R. M. Brown, S. Shankar, B. W. Lovett, A. Ardavan, T. Schenkel, E. E. Haller, J. W. Ager, S. A. Lyon, Solid-state quantum memory using the ^{31}P nuclear spin. *Nature* **455**, 1085–1088 (2008).

12. A. Abragam, *The Principles of Nuclear Magnetism* (Oxford Univ. Press, 1961).

13. M. I. D'yakonov, V. I. Perel', Cooling of a system of nuclear spins following optical orientation of electrons in semiconductors, *Zh. Eksp. Teor. Fiz.* **68**, 1514 (1975) [*Sov. Phys. JETP* **41**, 759 (1975)].

14. M. Goldman, *Spin Temperature and Nuclear Magnetic Resonance in Solids* (Clarendon Press, 1970).

15. I. A. Merkulov, Phase transition in a nuclear spin system in a semiconductor with optically oriented electrons, *Zh. Eksp. Teor. Fiz.* **82**, 319 (1982) [*Sov. Phys. JETP* **55**, 188 (1982)].

16. I. A. Merkulov, Y. I. Papava, V. V. Ponomarenko, S. I. Vasiliev, Monte Carlo simulation and theory in Gaussian approximation of a phase transition in the nuclear spin system of a solid. *Can. J. Phys.* **66**, 135 (1987).

17. I. A. Merkulov, Formation of a nuclear spin polaron under optical orientation in GaAs-type semiconductors. *Phys. Solid State* **40**, 930–933 (1998).

18. M. Vladimirova, D. Scalbert, M. S. Kuznetsova, K. V. Kavokin, Electron-induced nuclear magnetic ordering in n-type semiconductors. *Phys. Rev. B* **103**, 205207 (2021).

19. A. S. Oja, O. V. Lounasmaa, Nuclear magnetic ordering in simple metals at positive and negative nanokelvin temperatures. *Rev. Mod. Phys.* **69**, 1–136 (1997).

20. M. I. D'yakonov, V. I. Perel', Dynamic self-polarization of nuclei in solids, *Pis'ma Zh. Eksp. Teor. Fiz.* **16**, 563 (1972) [*JETP Lett.* **16**, 398 (1972)].

21. R. W. Mocek, V. L. Korenev, M. Bayer, M. Kotur, R. I. Dzhioev, D. O. Tolmachev, G. Cascio, K. V. Kavokin, D. Suter, High-efficiency optical pumping of nuclear polarization in a GaAs quantum well. *Phys. Rev. B* **96**, 201303 (2017).

22. G. Lampel, Nuclear dynamic polarization by optical electronic saturation and optical pumping in semiconductors. *Phys. Rev. Lett.* **20**, 491–493 (1968).

23. R. E. Worsley, N. J. Traynor, T. Grevatt, R. T. Harley, Transient linear birefringence in GaAs quantum wells: Magnetic field dependence of coherent exciton spin dynamics. *Phys. Rev. Lett.* **76**, 3224–3227 (1996).

24. D. R. Yakovlev, M. Bayer, Coherent spin dynamics of carriers in *Spin Physics in Semiconductors*, M. I. Dyakonov, Ed. (Springer International Publishing AG, 2017), chap. 6, pp. 155–206.

25. M. J. Snelling, G. P. Flinn, A. S. Plaut, R. T. Harley, A. C. Tropper, R. Eccleston, C. C. Phillips, Magnetic g factor of electrons in GaAs/$Al_xGa_{1-x}As$ quantum wells. *Phys. Rev. B* **44**, 11345–11352 (1991).

26. E. B. Aleksandrov, V. S. Zapasskii, Spin noise spectroscopy. *J. Phys. Conf. Ser.* **324**, 012002 (2011).

27. J. Baugh, Y. Kitamura, K. Ono, S. Tarucha, Large nuclear Overhauser fields detected in vertically coupled double quantum dots. *Phys. Rev. Lett.* **99**, 096804 (2007).

28. A. I. Tartakovskii, T. Wright, A. Russell, V. I. Fal'ko, A. B. Van'kov, J. Skiba-Szymanska, I. Drouzas, R. S. Kolodka, M. S. Skolnick, P. W. Fry, A. Tahraoui, H.-Y. Liu, M. Hopkinson, Nuclear spin switch in semiconductor quantum dots. *Phys. Rev. Lett.* **98**, 026806 (2007).

29. M. Vladimirova, S. Cronenberger, D. Scalbert, I. I. Ryzhov, V. S. Zapasskii, G. G. Kozlov, A. Lemaître, K. V. Kavokin, Spin temperature concept verified by optical magnetometry of nuclear spins. *Phys. Rev. B* **97**, 041301 (2018).

30. D. Paget, G. Lampel, B. Sapoval, V. I. Safarov, Low-field electron-nuclear spin coupling in gallium arsenide under optical pumping conditions. *Phys. Rev. B* **15**, 5780–5796 (1977).

31. V. M. Litvyak, R. V. Cherbunin, V. K. Kalevich, K. V. Kavokin, Local field of spin-spin interactions in the nuclear spin system of *n*-GaAs. *Phys. Rev. B* **108**, 235204 (2023).

32. M. Vladimirova, S. Cronenberger, A. Colombier, D. Scalbert, V. M. Litvyak, K. V. Kavokin, A. A. Lemaître, Simultaneous measurements of nuclear spin heat capacity, temperature and relaxation in GaAs microstructures. *Phys. Rev. B* **105**, 155305 (2022).

33. B. F. Gribakin, V. M. Litvyak, M. Kotur, R. André, M. Vladimirova, D. R. Yakovlev, K. V. Kavokin, Nuclear spin relaxation mediated by donor-bound and free electrons in wide CdTe quantum wells. *Phys. Rev. B* **109**, 195302 (2024).

34. S. Matsuki, "Dynamic nuclear self-polarization (DYNASP) of In in semiconductor InP," *JAERI Conference Proceeding* (Japan Atomic Energy Research Institute, 2005), pp. 123–127.

35. M. Koizumi, J. Goto, S. Matsuki, S. Nakamura, Dynamic nuclear self-polarization for measurements of nuclear magnetic moments. *Nucl. Instrum. Methods Phys. Res., Sect. B* **317**, 689–692 (2013).

36. V. L. Korenev, Dynamic self-polarization of nuclei in low-dimensional systems, *Pis'ma Zh. Eksp. Teor. Fiz.* **70**, 124 (1999) [*JETP Lett.* **70**, 129 (1999)].

37. V. L. Korenev, Nuclear spin nanomagnet in an optically excited quantum dot. *Phys. Rev. Lett.* **99**, 256405 (2007).

38. M. S. Rudner, L. S. Levitov, Self-polarization and dynamical cooling of nuclear spins in double quantum dots. *Phys. Rev. Lett.* **99**, 036602 (2007).

39. M. Kotur, F. Saeed, R. W. Mocek, V. L. Korenev, I. A. Akimov, A. S. Bhatti, D. R. Yakovlev, D. Suter, M. Bayer, Single-beam resonant spin amplification of electrons interacting with nuclei in a GaAs/(Al,Ga)As quantum well. *Phys. Rev. B* **98**, 205304 (2018).

40. M. Eickhoff, B. Lenzmann, G. Flinn, D. Suter, Coupling mechanisms for optically induced NMR in GaAs quantum wells. *Phys. Rev. B* **65**, 125301 (2002).

41. W. Hanle, Über magnetische Beeinflussung der Polarisation der Resonanzfluoreszenz. *Z. Phys.* **30**, 93–105 (1924).

42. M. I. D'yakonov, V. I. Perel', V. L. Berkovits, V. I. Safarov, Optical effects due to polarization of nuclei in semiconductors, *Zh. Eksp. Teor. Fiz.* **67**, 1912 (1974) [*Sov. Phys. JETP* **40**, 950 (1975)].

43. V. K. Kalevich, V. D. Kulkov, V. G. Fleisher, Onset of a nuclear polarization front due to optical spin orien tation in a semiconductor, *Pis'ma Zh. Eksp. Teor. Fiz.* **35**, 17 (1982) [*JETP Lett.* **35**, 20 (1982)].

44. M. Kotur, R. I. Dzhioev, K. V. Kavokin, V. L. Korenev, B. R. Namozov, P. E. Pak, Y. G. Kusrayev, Nuclear spin relaxation mediated by Fermi-edge electrons in n-type GaAs. *JETP Lett.* **99**, 37–41 (2014).

45. V. K. Kalevich, V. L. Korenev, O. M. Fedorova, Optical polarization of nuclei in GaAs/AlGaAs quantum well structures, *Pis'ma Zh. Eksp. Teor. Fiz.* **52**, 964 (1990) [*JETP Lett.* **52**, 349 (1990)].

46. E. A. Chekhovich, A. Ulhaq, E. Zallo, F. Ding, O. G. Schmidt, M. S. Skolnick, Measurement of the spin temperature of optically cooled nuclei and GaAs hyperfine constants in GaAs/AlGaAs quantum dots. *Nat. Mater.* **16**, 982–986 (2017).

47. I. A. Merkulov, V. G. Fleisher, "Optical orientation of the coupled electron-nuclear spin system of a semiconductor," in *Optical Orientation*, F. Meier, B. P. Zakharchenya, Eds. (North-Holland, 1984), chap. 5, pp. 137–207.

48. V. K. Kalevich, K. V. Kavokin, I. A. Merkulov, M. R. Vladimirova, "Dynamic nuclear polarization and nuclear fields," in *Spin Physics in Semiconductors*, M. I. Dyakonov, Ed. (Springer International Publishing AG, 2017), chap. 12, pp. 387–430.
